# Safety Issues in the Development of an Innovative Medical Parallel Robot Used in Renal Single-Incision Laparoscopic Surgery

**DOI:** 10.3390/jcm12144617

**Published:** 2023-07-11

**Authors:** Doina Pisla, Nicolae Crisan, Bogdan Gherman, Iulia Andras, Paul Tucan, Corina Radu, Alexandru Pusca, Calin Vaida, Nadim Al Hajjar

**Affiliations:** 1 Research Center for Industrial Robots Simulation and Testing—CESTER, Technical University of Cluj-Napoca, 400114 Cluj-Napoca, Romania; doina.pisla@mep.utcluj.ro (D.P.); alexandru.pusca@mep.utcluj.ro (A.P.); calin.vaida@mep.utcluj.ro (C.V.); 2 Department of Urology, “Iuliu Hatieganu” University of Medicine and Pharmacy, 400012 Cluj-Napoca, Romania; nicolae.crisan@umfcluj.ro (N.C.); iulia.andras@umfcluj.ro (I.A.); na.hajjar@umfcluj.ro (N.A.H.); 3 Department of Internal Medicine, “Iuliu Hatieganu” University of Medicine and Pharmacy, 400012 Cluj-Napoca, Romania; corina.radu@umfcluj.ro

**Keywords:** single-incision laparoscopic surgery, risk assessment, parallel robot, design, safety

## Abstract

Robotic-assisted single-incision laparoscopic surgery (SILS) is becoming an increasingly widespread field worldwide due to the benefits it brings to both the patient and the surgeon. The goal of this study is to develop a secure robotic solution for SILS, focusing specifically on urology, by identifying and addressing various safety concerns from an early design stage. Starting with the medical tasks and protocols, the technical specifications of the robotic system as well as potential; hazards have been identified. By employing competitive engineering design methods such as Analytic Hierarchy Process (AHP), Risk assessment, and Failure Mode and Effects Analysis (FMEA), a safe design solution is proposed. A set of experiments is conducted to validate the proposed concept, and the results strongly support the development of the experimental model. The Finite Element Analysis (FEA) method is applied to validate the mechanical architecture within a set of simulations, demonstrating the compliance of the robotic system with the proposed technical specifications and its capability to safely perform SILS procedures.

## 1. Introduction

Laparoscopic surgery is recognized as one of the most significant advancements of the 20th century, with the initial laparoscopic procedure being performed in the early 1980s. Since then, laparoscopic surgery has undergone continuous evolution and emerged as an alternative to traditional surgery, which entails large incisions leading to prolonged recovery periods, postoperative pain, substantial blood loss, and unfavorable unpleasant cosmetic outcomes, characterized by highly visible scars [1,2,3].

Numerous laparoscopic methods and techniques have been developed, with some of the most renowned including Minimally Invasive Surgery (MIS), single-incision laparoscopic surgery (SILS), and Natural Orifice Transluminal Endoscopic Surgery (NOTES) [4,5].

MIS is a technique which was introduced and has been used since the early 1980s. It involves a limited number of incisions, resulting in less postoperative trauma and shorter hospitalization periods [6,7]. In 1985, Erich Mühe conducted the first laparoscopic cholecystectomy, which brought attention to a range of benefits, including decreased recovery time, a minimal number of incisions, reduced postoperative trauma, and diminished blood loss during the procedure [6,7,8,9].

Due to the promising results obtained with MIS and the aim of minimizing the number of incisions, in 1997, a single incision made at the level of the umbilicus was first employed in general surgery. This incision served as the entry point for all the instruments, introducing a new technique known as single-incision laparoscopic surgery (SILS), which emerged as a promising alternative to MIS [10,11,12,13].

However, SILS also presents a range of disadvantages and limitations, which are extensively discussed in [14,15]. These include reduced workspace, low ergonomics and dexterity, the triangulation effect of instruments, and surgeon’s hand tremors. To address these drawbacks and enhance single-incision laparoscopic surgery, several robotic systems have been developed and described in [16,17,18,19,20,21,22,23].

The first robotic system utilized in SILS was the da Vinci S in 2008 [24], employing two standard instruments and an endoscopic camera inserted in the central multilumen port. The surgical approach proved highly successful at the time, mitigating issues such as hand tremors, limited dexterity, precision, postoperative pain, and ergonomic challenges for the surgeon [19]. Building upon these achievements, in 2018, the Intuitive Surgical company developed the da Vinci SP, the first dedicated robotic system specifically designed for SILS. This system has a dedicated trocar (25 mm) through which the two active articulated instruments (7-DOF/instrument) and the articulated 3D HD endoscopic camera were inserted. The da Vinci SP robot introduced notable improvements in terms of precision, accuracy, 3D visualization provided by the articulated endoscopic camera, motion scaling, and tremor elimination compared with the da Vinci S [25,26].

Another robotic system utilized in SILS is the Senhance system, developed by TransEnterix Surgical [27]. Senhance [27] stands out as the first surgical robotic system to implement haptic force feedback for enhanced control, enabling the surgeon to perceive tissue stiffness. The console of the robot consists of a 3D HD monitor, specialized 3D glasses, an eye-tracking camera, two master laparoscopic controllers and a control pedal for instrument energy activation [27]. All these systems are operated using a master–slave architecture described in detail in [18,28], enabling the surgeon to manipulate the robotic structure remotely and eliminating disadvantages such as hand tremors, suboptimal surgeon ergonomics, limited precision, and dexterity.

Nowadays, SILS robotic systems adopt two instrument configuration modes: the X configuration and the Y configuration. The X configuration provides a broader intraoperative space and overcomes the triangulation effect of non-robotic systems. However, a notable drawback of this configuration is the potential for external collisions between the robotic arms. The Y configuration helps mitigate collisions between the instruments by utilizing curved instruments or modules that allow shape alteration. Nevertheless, the main disadvantage of this technique is the very low payload capacity of the instruments and a more restricted workspace compared to the X configuration. A comprehensive analysis of the X and Y configurations is described in [8,24], while an extensive examination of the advantages and limitations of employing robotic systems in SILS can be found in [18,29,30,31,32].

While although several commercial solutions for robotic-assisted SILS have been previously developed, further research is necessary to develop more accurate and cost-effective tools, making robotic solutions more affordable for hospitals and clinics. While cost-effectiveness is important, ensuring patient safety is of utmost importance. The paper focuses on the development of a safe robotic solution for SILS employing various engineering methods and analyses to address safety concerns from the early stages of design.

**Figure 1:**
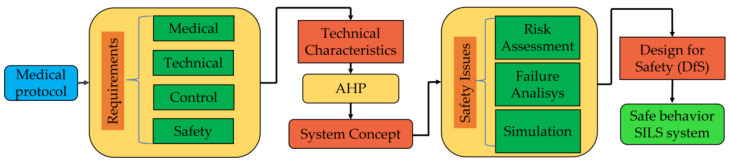
The design for safety approach for a robotic system for SILS (AHP—Analytical Hierarchy Process is a technique used to prioritize the main technical characteristics of the robotic structure, which is based on the direct comparison of the technical characteristics by assigning an importance score between 1/9 (the least important characteristic) and 9 (most important feature). Design for Safety (DfS) integrates all the results obtained from risk analysis, failure analysis and simulation into the design of the robot).

To assess the safety of the robotic system, the authors propose the following path (Figure 1): firstly, the medical protocol is analyzed, and the medical, technical, control and safety requirements are identified. These requirements are then defined as technical characteristics and prioritized using competitive engineering methods. The prioritization method outcome is used to define the initial concept of the robotic system, encompassing mechanical design and control system.

Once the initial concept is developed, an analysis is conducted to identify safety issues. This process includes a risk assessment process to identify potential risks associated with the robotic-assisted SILS procedure, an FMEA to determine the failure modes of the system and a series of simulations to determine system functionality. After identifying safety issues, design solutions aimed at mitigating them are proposed, leading to enhancements in the mechanical architecture, control, and sensor system.

The paper is structured as follows: Section 2 presents the Materials and Methods used to develop the concept of the robotic system (AHP: Analytic Hierarchy Process, Risk-Assessment; FMEA: Failure Modes and Effects Analysis, experimental setup). Section 3 contains the results obtained by applying the methods presented in the last section. The results imply the design of the robotic system and a series of simulations that validate the proposed architecture. The discussion and conclusions are presented in Section 4 and Section 5.

## 2. Materials and Methods

### 2.1. Identification and Prioritization of Technical Characteristics for a Parallel Robotic Structure Used in SILS

The main technical characteristics (TCs) of a medical robot for SILS are presented in Table 1. The TCs have been further prioritized using the Analytical Hierarchical Process (AHP), where each TC’s importance is assessed in relation to the others, assigning values between 4 and 1/3 (4 representing the value for the most important characteristic and 1/3 for the least important characteristic).

**Figure 2:**
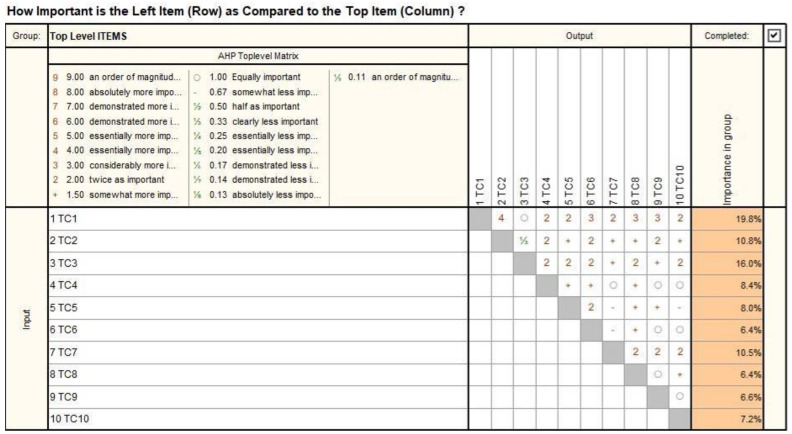
Prioritization of technical characteristics using the AHP matrix (prioritization of the technical characteristics was carried out by directly comparing the technical characteristics with each other (line with column), assigning numerical values for each individual position, e.g.,: Technical Characteristic 1 (TC1) was compared in turn with each characteristic-TC2–TC10, revealing the degree of importance presented in the form of a percentage).

**Figure 3:**
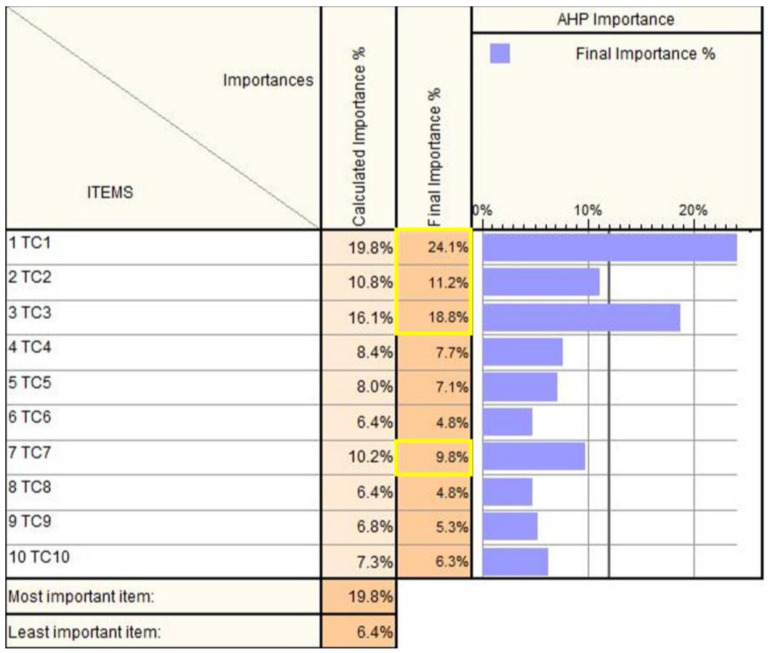
AHP results of technical characteristics of the PARA–SILSROB structure.

The AHP matrix is presented in Figure 2, and the results (the TCs prioritization) are presented in Figure 3.

The most important technical characteristics are TC1 (24.1%), TC3 (18.8%), TC2 (11.2%), and TC7 (9.8%). These technical characteristics primarily emphasize the safety of the robotic system during operation, the workspace, the ability to remove the robot and medical instruments in case of an emergency, and the implementation of a control system based on a master–slave architecture [18,33].

### 2.2. The PARA-SILSROB Robotic System

**Figure 4:**
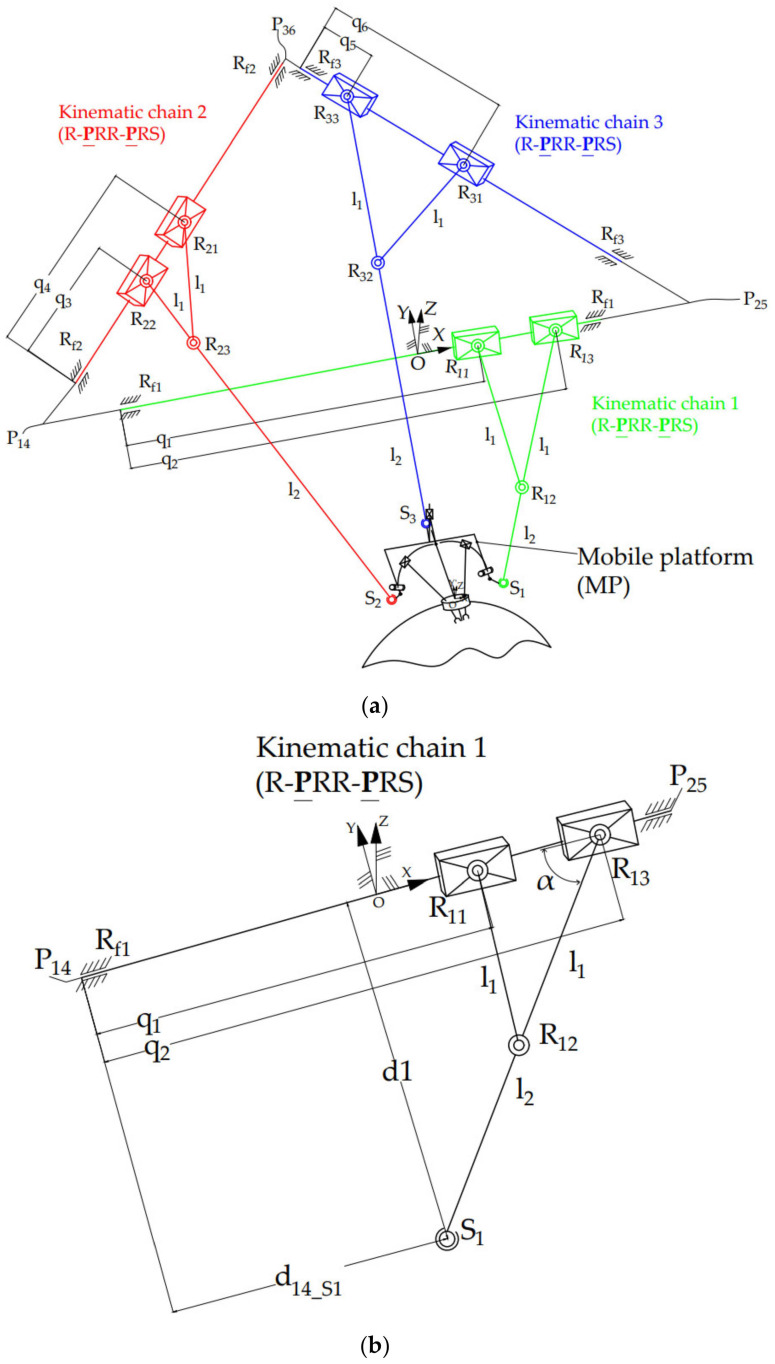

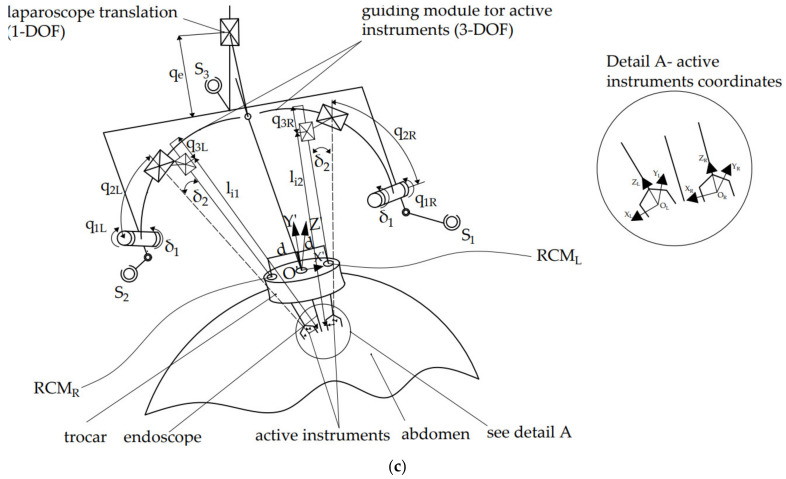
The PARA-SILSROB robotic system architecture: (**a**) the PARA-SILSROB kinematic scheme; (**b**) architecture of the three identical kinematic chains of PARA-SILSROB; (**c**) mobile platform detail.

Based on technical requirements and in compliance with the medical protocol presented in [34] and summarized below, the concept of a robotic system for SILS consists of the following modules:

➢ A 6 Degrees of Freedom (DOF) parallel robot (Figure 4a) guiding a mobile platform (MP)—Figure 4a.
➢ An endoscopic camera module having 1-DOF designed to perform the laparoscope insertion into the patient’s body—Figure 4c.
➢ Two serial modules, each having 3-DOF and designed to guide the active instruments for SILS—Figure 4c. The instrument modules are designed to work in the X configuration.

The medical SILS protocol with respect to the proposed robot consists of the following steps:

- Preparation: patient anesthesia, operating room preparation, position the robot to the SILS port trocars (where the Artificial Intelligence module developed in [35] can be used), save the laparoscope RCM and find the *RCM_L_* and *RCM_R_* positions using the 6-DOF parallel robot;
- Instruments insertion: the three instruments are placed in the surgical field. Usually, for the vast majority of SILS procedures (including liver cancer—hepatocellular carcinoma—or stomach cancer), the incision and insertion of the SILS port are performed at the level of the umbilicus, with the patient positioned on his back. However, there may be situations where the incision is made retroperitoneally below the 12th rib, with the patient positioned on one side (in the case of kidney surgeries). The maximum orientation of the mobile platform after inserting the endoscopic camera and active instruments into the patient’s body is limited to ±30° [22]. The endoscopic camera is inserted into the patient’s body approximately 150 mm, and the maximum insertion depth of the active instruments is 270 mm [28,36].
- MP positioning towards the targeted lesion: the amplitude of the movements of the modules attached to the mobile platform is limited to ±45° [29].
- Surgical task: the MP is locked in position, and the two modules guiding the active instruments perform individual tasks, remotely guided by the surgeon. It becomes obvious that if the laparoscope requires further orientation, which is performed with the MP, the *RCM_L_* and *RCM_R_* positions will be altered, and a compensation motion will be from the two modules is required.

The 6-DOF parallel robot performs the position and orientation of the MP, on which the other guiding modules (for the laparoscope and active instruments) are positioned. The Remote Center of Motion (RCM) [37] of the 6-DOF parallel robot is set for the laparoscope. This means that the laparoscope’s orientation during the SILS procedure is performed by orienting the MP of the 6-DOF robot.

The fixed coordinates system OXYZ is placed in the horizontal plane, which contains the *R_fi_*, *i* = 1.3 revolute joints, in the middle of the *KC*_1_ kinematic chain. *KC*_1_ (Figure 4b) is actuated by the prismatic joints *q*_1_ and *q*_2_ and has four passive revolute joints, namely: *R*_11_*, R*_12_*, R*_13_ and *R_f_*_1_ and a passive spherical joint, *S*_1_. Thus, the *KC*_1_ has three DOF: two active DOF (*q*_1_*, q*_2_) and a passive one: the revolute joint *R_f1_*, which allows the *S_1_* spherical joint to perform a free motion around the *P*_14_ *P*_25_ axis. The other kinematic chains (*KC*_2_ and *KC*_3_) have an identical architecture to *KC*_1_.

The MP consists of the laparoscopic module with a 1-DOF prismatic joint and the two guiding modules of the active instruments with 3-DOF. The two modules have serial architecture (RPP), with three active joints: a revolute joint, namely *q*_1*L*_ for the left module and, respectively, *q*_1*R*_ for the right module, and two active prismatic joints, namely *q*_2*L*_ and *q*_3*L*_ for the left module and, respectively *q*_2*R*_ and *q*_3*R*_ for the right module. The *q*_2*L*_ and *q*_2*R*_ trajectories are curvilinear, with the circle center being the RCM of the two instruments [38], architecturally constrained.

### 2.3. Inverse Kinematics of the PARA-SILSROB Robotic Structure

Inverse kinematics is used within the control system of PARA-SILSROB to determine the active joint values required to reach the desired position of the SILS instruments.

Regarding the 6-DOF parallel robot, which performs the MP position and orientation, the geometric parameters (*l*_1_, *l*_2_, *l_PM_*, *l_FP_*) of the robotic structure (Figure 4a) and the six independent parameters of the endoscopic camera *X_E_*, *Y_E_*, *Z_E_* (the tip coordinates in the OXYZ coordinate system) and its orientation *ψ*, *θ*, *φ* (the Euler angles) are input data. The active joints coordinates (*q*_1_, *q*_2_, *q*_3_, *q*_4_, *q*_5_, *q*_6_) are determined using:

(1) $$\left\{\begin{array}{l} q_{1}=q_{2}-2\cdot l_{1}\cdot \cos\left(\alpha_{12}\right)\\ q_{2}=\sqrt{{\left(l_{1}+l_{2}\right)}^{2}-d_{1}^{2}}+d_{14\_S1}\\ q_{3}=q_{4}-2\cdot l_{1}\cdot \cos\left(\alpha_{34}\right)\\ q_{4}=\sqrt{{\left(l_{1}+l_{2}\right)}^{2}-d_{2}^{2}}+d_{36\_S2}\\ q_{5}=q_{6}-2\cdot l_{1}\cdot \cos\left(\alpha_{56}\right)\\ q_{6}=\sqrt{{\left(l_{1}+l_{2}\right)}^{2}-d_{3}^{2}}+d_{25\_S3} \end{array}\right.$$

where *d*_1_, *d*_2_, and *d*_3_ represents the distance between the *S_i_* points and the triangular frame of the robotic structure, α_12_, α_34_ and α_56_ is the angle between the robotic arms and the active translational joints and *d*_14*_S*1_, *d*_36*_S*2_ and *d*_25*_S*3_ is the distance between the kinematic chains (*P*_14_, *P*_36_, *P*_25_) and the spherical joints (*S*_1_, *S*_2_, *S*_3_). An extensive study regarding the inverse geometric model was presented in [29].

Regarding the MP modules, the input parameters for the geometric model are the coordinates of the endoscopic camera (*RCM_E_*) and the coordinates of the tip of the active instruments and endoscopic camera (*O_L_*, *O_E_*, *O_R_*). The output is the values of the three modules (endoscopic camera −*q_e_*; module left −*q*_1*L*_, *q*_2*L*_, *q*_3*L*_ and the right module −*q*_1*R*_, *q*_2*R*_, *q*_3*R*_).

The input parameters are:

(2) $$\left\{\begin{array}{l} RCM_{E}={\left[X_{RCM_{E}}\;Y_{RCM_{E}}\;Z_{RCM_{E}}\right]}^{T},\\ O_{instL}={\left[X_{instL}\;Y_{instL}\;Z_{instL}\right]}^{T},\\ O_{E}={\left[X_{E}\;Y_{E}\;Z_{E}\right]}^{T},\\ O_{instR}={\left[X_{instR}\;Y_{instR}\;Z_{instR}\right]}^{T} \end{array}\right.$$

Considering that the distance *RCM_L_ − RCM_E_ = RCM_L_ − RCM_E_ = d*, and that the *RCM_L_*, *RCM_E_* and *RCM_R_* are collinear, the position of the RCMs for modules used to handle active instruments is:

(3) $$\left\{\begin{array}{l} RCM_{L}={\left[X_{RCM_{E}}-d\left(1-\cos\left(\theta \right)\right)\;Y_{RCM_{E}}\;Z_{RCM_{E}}-d\left(1-\sin\left(\theta \right)\right)\right]}^{T},\\ RCM_{R}={\left[X_{RCM_{E}}+d\left(1-\cos\left(\theta \right)\right)\;Y_{RCM_{E}}\;Z_{RCM_{E}}+d\left(1-\sin\left(\theta \right)\right)\right]}^{T} \end{array}\right.$$

The coordinates of the active instrument relative to the module left with respect to the rotation angle *δ*_1_ are:

(4) $$\left\{\begin{array}{l} X_{origL}=\left(X_{instL}+d-X_{RCM_{E}}\right)\cdot \cos\left(\delta_{1}\right)+\left(Y_{RCM_{E}}-Y_{instL}\right)\cdot \sin\left(\delta_{1}\right)\\ Y_{origL}=\left(X_{instL}+d-X_{RCM_{E}}\right)\cdot \sin\left(\delta_{1}\right)+\left(Y_{instL}-Y_{RCM_{E}}\right)\cdot \cos\left(\delta_{1}\right)+Y_{RCM_{E}}\\ Z_{origL}=Z_{instL} \end{array}\right.$$

The coordinate of the active instrument relative to the module right with respect to the rotation angle *δ*_1_ are:

(5) $$\left\{\begin{array}{l} X_{origR}=\left(X_{instR}-d-X_{RCM_{E}}\right)\cdot \cos\left(-\delta_{1}\right)+\left(Y_{RCM_{E}}-Y_{instR}\right)\cdot \sin\left(-\delta_{1}\right)\\ Y_{origR}=\left(X_{instR}-d-X_{RCM_{E}}\right)\cdot \sin\left(-\delta_{1}\right)+\left(Y_{instR}-Y_{RCM_{E}}\right)\cdot \cos\left(-\delta_{1}\right)+Y_{RCM_{E}}\\ Z_{origR}=Z_{instR} \end{array}\right.$$

Based on Equations (4) and (5), the orientation angles of the left and right modules with respect to the RCM of each instrument can be expressed as follows:

(6) $$\left\{\begin{array}{l} \theta_{instL}=\mathrm{sgn}\left(Z_{RCM_{L}}-Z_{origL}\right)\cdot \mathrm{sgn}\left(-X_{origL}\right)\cdot \mathrm{atan}2\left(\left|X_{origL}\right|,\left|Z_{RCM_{L}}-Z_{origL}\right|\right)\\ \psi_{instL}=\mathrm{sgn}\left(Z_{RCM_{L}}-Z_{origL}\right)\cdot \mathrm{sgn}\left(Y_{RCM_{L}}-Y_{origL}\right)\cdot \mathrm{atan}2\left(\left|Y_{RCM_{L}}-Y_{origL}\right|,\frac{\left|Z_{RCM_{E}}-Z_{origL}\right|}{\cos\left(\theta_{instL}\right)}\right)\\ ins_{instL}=\mathrm{sgn}\left(Z_{RCM_{L}}-Z_{origL}\right)\cdot \sqrt{X_{origL}{}^{2}+{\left(Y_{RCM_{L}}-Y_{origL}\right)}^{2}+{\left(Z_{RCM_{L}}-Z_{origL}\right)}^{2}} \end{array}\right.$$

(7) $$\left\{\begin{array}{l} \theta_{instR}=\mathrm{sgn}\left(Z_{RCM_{R}}-Z_{instR}\right)\cdot \mathrm{sgn}\left(-X_{instR}\right)\cdot \mathrm{atan}2\left(\left|X_{instR}\right|,\left|Z_{RCM_{R}}-Z_{instR}\right|\right)\\ \psi_{instR}=\mathrm{sgn}\left(Z_{RCM_{R}}-Z_{instR}\right)\cdot \mathrm{sgn}\left(Y_{RCM_{R}}-Y_{instR}\right)\cdot \mathrm{atan}2\left(\left|Y_{RCM_{R}}-Y_{instR}\right|,\frac{\left|Z_{RCM_{E}}-Z_{instR}\right|}{\cos\left(\theta_{instR}\right)}\right)\\ ins_{instR}=\mathrm{sgn}\left(Z_{RCM_{R}}-Z_{instR}\right)\cdot \sqrt{X_{instR}{}^{2}+{\left(Y_{RCM_{R}}-Y_{instR}\right)}^{2}+{\left(Z_{RCM_{R}}-Z_{instR}\right)}^{2}} \end{array}\right.$$

The active coordinates for the left and right modules in the mobile coordinate system attached to the MP and placed in the RCM of the laparoscope (O’X’Y’Z’) are expressed based on the equations presented above, obtaining the following equations:

(8) $$\left\{\begin{array}{l} q_{1L}=\theta_{instL}+\delta_{2}\\ q_{2L}=R_{L}\cdot \left(\psi_{L}+\frac{\pi }{2}\right)\\ q_{3L}=\left(R_{L}-n_{ins}\right)+ins_{instL} \end{array}\right.,\left\{\begin{array}{l} q_{1R}=\theta_{instR}+\delta_{2}\\ q_{2R}=R_{R}\cdot \left(\psi_{R}+\frac{\pi }{2}\right)\\ q_{3R}=\left(R_{R}-n_{ins}\right)+ins_{instR} \end{array}\right.$$

where *R_L_*, *R_R_* represents the radius of each module, *n_ins_* represent the minimum insertion length of the active instrument and *ins_L_*, *ins_R_* represents the length of the active instruments.

### 2.4. Design and Functionality of the PARA-SILSROB Robotic Structure

**Figure 5:**
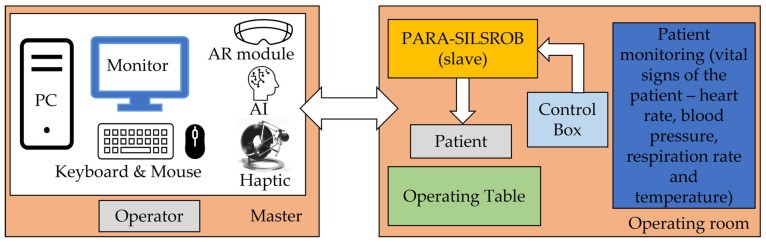
The master–slave architecture of PARA-SILSROB.

The robotic system is designed employing a master–slave architecture (Figure 5) where the operator is positioned at the master console and has access to various tools to control the robotic system. The operator can utilize the keyboard and mouse to prepare the system procedure, importing data such as personal information, evaluation charts or examination data. Additionally, haptic devices enable the operator to manipulate the robot during the operation and simulations.

The system is equipped with an augmented reality module, which is utilized both in the operation and preplanning. This module, based on the AI agent segmentation algorithm described in [35,39]) allows the medical professional to simulate the entire procedure and verify if the robotic system can reach the target organ or specific points within the organ. The entire process is supervised by a master computer that facilitates communication with visualization tools, input devices, haptic devices, and patient monitoring devices.

**Figure 6:**
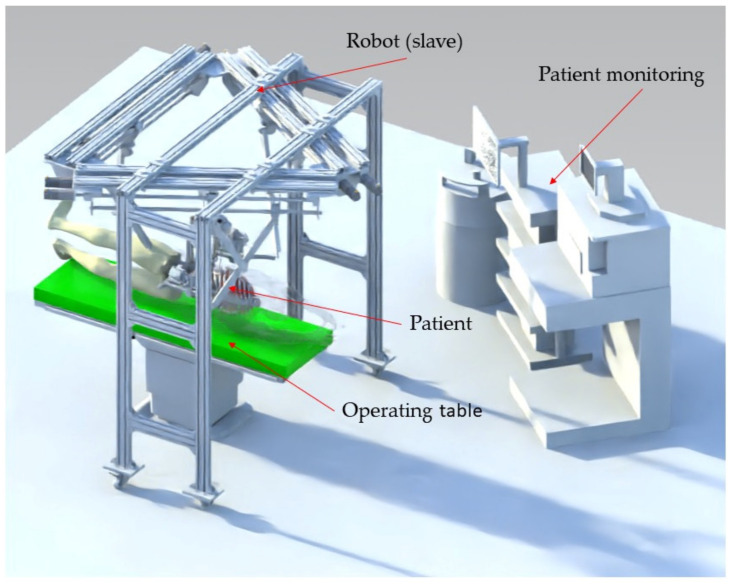
PARA-SILSROB integrated in the operating environment.

The control box contains a powerful Programmable Logical Computer (PLC) capable of sending and receiving real-time commands. It also manages the signals from the sensors mounted on the robotic structure and sends them to the master computer for further processing. The slave robot executes the required motions transmitted to the control box through the haptic device and master computer. The robot is equipped with sensors that monitor the position of the main mechanical components during its operation. Figure 6 presents the integration of the robotic system within the operating room environment.

**Figure 7:**
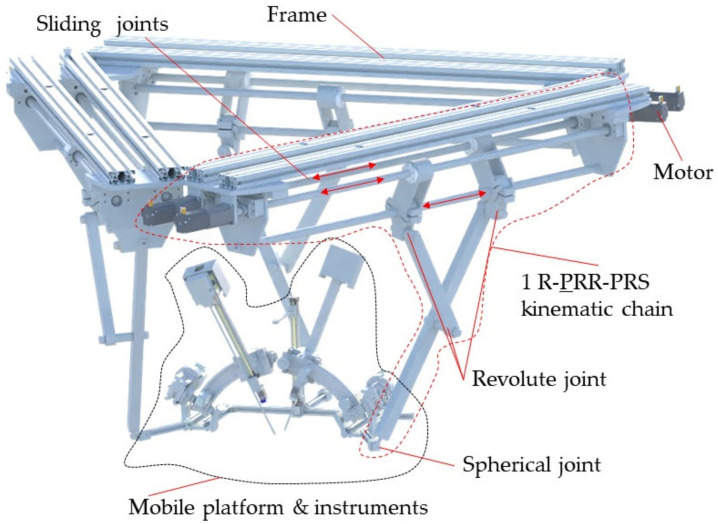
The structure of the PARA-SILSROB robot with mobile platform.

The architecture of the PARA-SILSROB robot [40] is shown in Figure 7, where the three identical kinematic R-PRR-PRS chains (*KC*_1_, *KC*_2_ and *KC*_3_) are connected to the fixed frame. The actuation of the kinematic chains is achieved through six motors. The connection of the kinematic chains to the mobile platform is achieved through passive spherical joints that are attached to the three main arms of the PARA-SILSROB robotic structure.

**Figure 8:**
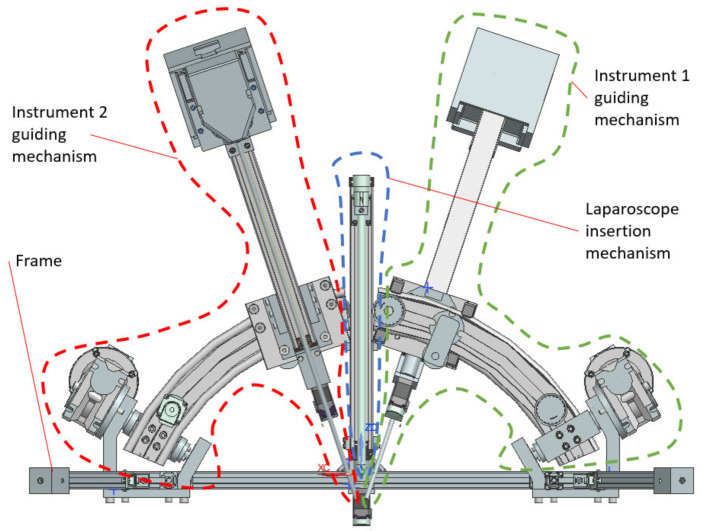
Mobile platform detail view with the modules for each instrument.

The mobile platform (Figure 8) consists of an aluminum frame to which the three modules for manipulating the instruments required for the medical procedure are attached. The central component of the mobile platform is the laparoscope insertion mechanism positioned in the center. It consists of a linear shaft, a servomotor, and the laparoscope attaching element. On both sides of the laparoscope insertion mechanism, the instrument guiding mechanisms are mounted. Each of these modules includes six servomotors capable of orienting and inserting the active instruments around a fixed point called the Remote Center of Motion (RCM) [37].

### 2.5. Risk Assessment of the PARA-SILSROB Robotic System

The International Organization for Standardization (ISO) defines risk as “an effect of uncertainty on objectives”, where uncertainty can be defined as a series of events that may or may not occur, thus causing ambiguity or a lack of information; a clear definition of risk is established in ISO 31000 (2009) and ISO 73:2002 [41,42].

To prevent and reduce the risks that may occur during the exploitation of the PARA-SILSROB robotic structure (Figure 4a), a risk analysis was generated based on the ISO 14971:2019 (Medical Device standard) standard [43].

**Figure 9:**
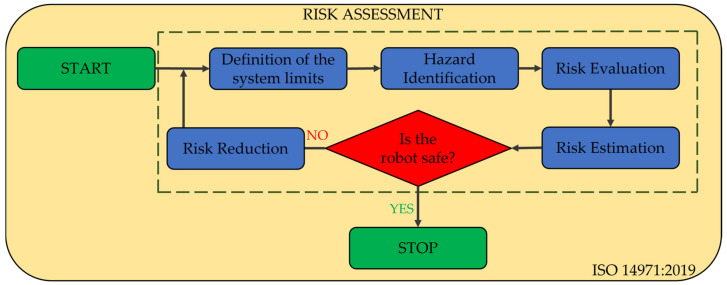
Risk analysis of the PARA-SILSROB robotic system.

According to Figure 9, the analysis is divided into five major steps:

- Definition of the system limits;
- Hazard identification;
- Risk estimation;
- Risk evaluation;
- Risk reduction.

A series of hazards have been identified and classified into mechanical, electrical, thermal, vibrations, noise, and ergonomic:

**Mechanical hazards:**
  - **MH1:** Collision between the guiding module of the laparoscope and the two active instruments. This hazard poses significant risks to the safety of the procedure, as the shocks recorded during this event may lead to mechanical damage, compromised placement accuracy, and control issues (i.e., due to sensory system physical failure).
  - **MH2:** Collision between the mobile platform and the patient body. During a collision between the MP and the patient, the patient’s tissue may undergo deformation and damage. This has a detrimental impact on the procedure safety as it can result in improper positioning of the laparoscope and active instruments due to the displacement of their RCMs.
  - **MH3:** Patient tissue may experience excessive strain due to the displacement of the Remote Center of Motion when orienting the endoscopic camera. As mentioned in Section 2.2, the orientation of the endoscopic camera is achieved using the PARA-SILSROB parallel robot, which possesses 6-DOF. Consequently, the architecturally constrained RCMs of the two modules guiding the SILS instruments will shift in accordance with the camera’s orientation angle. Section 2.7 presents an experimental setup designed to evaluate the risk of the RCM displacement for the instruments during the SILS procedure.
**Electrical hazards:**
  - **EH1:** Risk of power outage.
  - **EH2:** Risk of electrocution of the patient. Improper grounding may lead to such risks.
  - **EH3:** Risk of short circuit.
  - **EH4:** Risk of sensors system malfunction.
  - **EH5:** Risk of communication protocol malfunction (cabling issues).
**Thermal hazard:**
  - **TH1:** Tissue burns caused by wrong manipulation of active instruments (Permanent Cautery Hook).
  - **TH2:** Burns caused by overheating of the active instruments (Permanent Cautery Hook).
**Vibration hazards:**
  - **VH1:** High level of tremor at the tip of the instruments (and the laparoscope) due to vibration modalities of the robotic system.
**Noise hazard:**
  - **NH1:** Acoustic discomfort caused by driving the motion of the mobile components when the robotic system is operated.

The subsequent stage in risk assessment involves estimating the level of risk, which can be accomplished through various methods such as utilizing a risk matrix, risk graph or numerical scoring. Risks can be estimated in two ways according to ISO 31000 (2009) and ISO 73:2002 [41,42,44], based on severity and probability of occurrence. To estimate the risk, the numerical scoring method [45] is used to estimate the risk, and the results obtained are presented in Section 3.

The risk severity can be numerically estimated by assigning scores between 0 and 100, with the score distribution as follows: catastrophic (100), severe (90–99), moderate (30–89) and minor (0–29).

The probability score can also be expressed numerically using the following notations: very likely (100), likely (70–99), unlikely (30–69) and remote (0–29).

The final score for each hazard is determined based on the arithmetic mean between the severity and probability scores.

To obtain an objective estimation of the identified risks, a questionnaire (Appendix A) was distributed to a number of 10 persons with backgrounds in medical application and engineering fields. These participants were asked to evaluate the PARA-SILSROB robotic system by completing two tables in the questionnaire with numerical values. Table A1 was used to record the severity of the risks, and Table A2 was used to record the probability. The results of the questionnaire are presented in Section 3.

The total score assigned to each identified hazard is classified according to the following value scale:

- **High**: over 151;
- **Medium**: 101–150;
- **Low**: 61–100;
- **Negligible**: 0–50.

The results of the questionnaires are presented in Table 2 (severity) and Table 3 (probability).

### 2.6. Failure Mode and Effects Analysis for the PARA-SILSROB System

To ensure the development of a safe robotic system, Failure Mode and Effects Analysis (FMEA) was employed to identify, reduce, and eliminate risks that may arise during the operation of the PARA-SILSROB robotic system. This analysis helped identify potential failure modes in the robot structure and understand the effects and possible causes that could lead to these failures, ultimately influencing the system’s safety. The aim of this analysis is to establish a series of actions that can effectively eliminate or mitigate these failures, starting with the highest priority ones. In Table 4, the FMEA of the PARA-SILSROB robotic structure is presented, outlining the main systems functions and potential failure modes. Additionally, a series of actions necessary to mitigate them have been identified.

### 2.7. Experimental Tests to Assess the Instruments RCM Displacement Risk (MH3)

**Figure 10:**
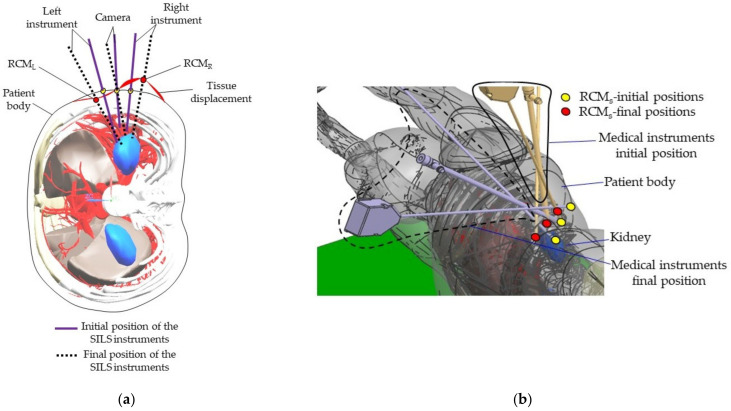
The SILS instruments RCMs displacement during the endoscopic camera orientation: (**a**) transversal section. (**b**) isometric view.

**Figure 11:**
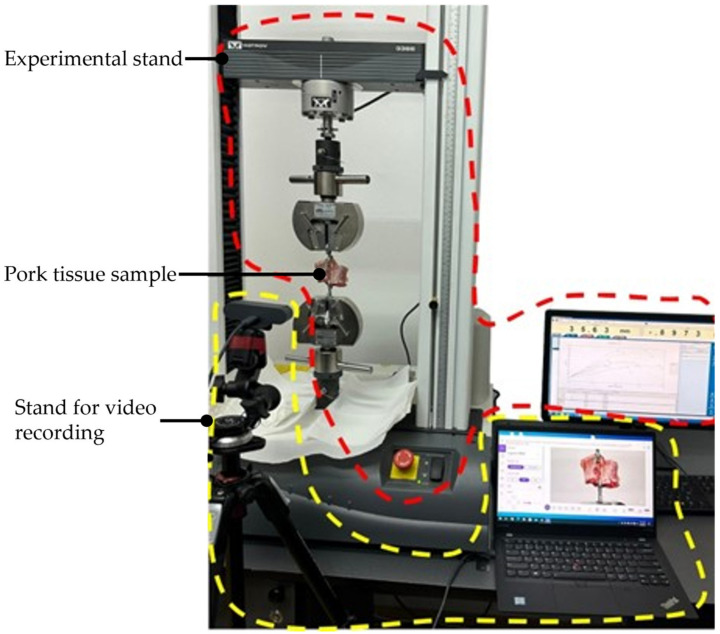
The intercostal muscles strain test bed.

**Figure 12:**
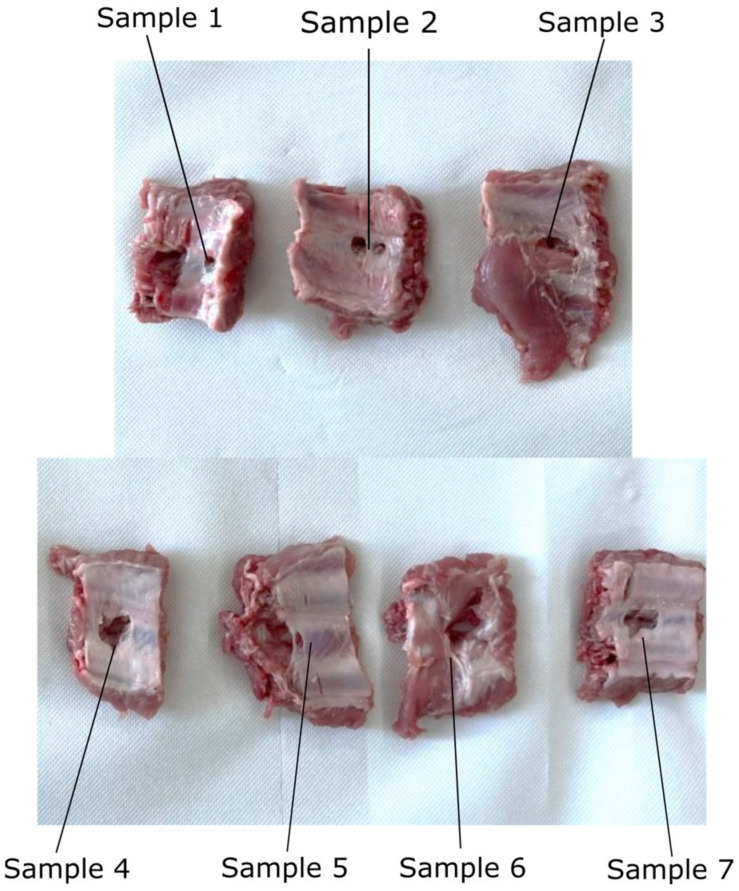
The pork intercostal muscles tested samples.

The endoscopic camera orientation will alter the RCM position of the two modules during the SILS procedure, as presented in Figure 10. A set of experimental tests have been performed to assess the intercostal muscle strain limits using pork tissue. The test bed is presented in Figure 11 and consists of: the Instron 3366 Universal Testing Machine [46], a monitoring camera (with PC) and pork tissue. Seven tissue samples from various areas of the pork’s body have been tested (Figure 12), where the pork tissues have been gripped using industrial steel hooks (with a diameter of Φ6 mm) between the machine’s grippers. The speed used for the experiment was 10 mm/min for samples 1–5 and 20 mm/min for samples 6–7. During the tests, the tissue samples were extended (stretching the intercostal muscles) until the registered force started to decrease, showing that the muscle fibers had broken (and the tissue was being detached from the ribs).

## 3. Results

### 3.1. Risk Reduction Solutions for the PARA-SILSROB System

**Figure 13:**
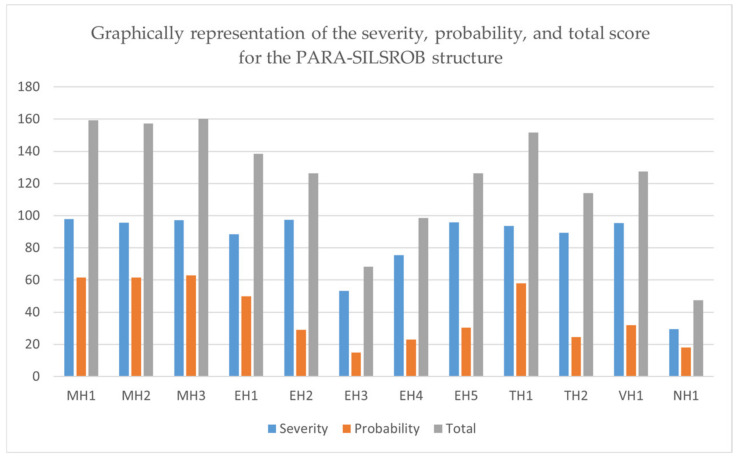
Graphical representation of the severity, probability, and total score for the PARA-SILSROB robotic structure.

The aforementioned analyses were generated to emphasize the safety characteristics by identifying, reducing, or eliminating the identified hazards that may occur while using the PARA-SILSROB robotic system. The hazards and failure modes were reduced and eliminated with the help of safety measures divided into two categories: control and mechanical components used to generate a safe operating solution. The scores obtained for each hazard are graphically represented in Figure 13.

The most severe hazard, according to Table 5, is MH1 (97.9), the most probable hazard is MH3 (63), and the highest overall score is identified for hazard MH3 (160.1).

The risk evaluation is presented in Table 5, and based on the results generated in this table, the risk reduction is performed for the hazards with the highest score (based on scale from Section 2).

According to the results presented in Table 5, the hazards that need reduction are the hazards with medium and high scores. Reducing these hazards is achieved in the final stage of this risk analysis, and the methods used to mitigate these hazards are presented in Table 6.

### 3.2. Results of FMEA for PARA-SILSROB System

This sub-section presents the results of the Failure Modes and Effects Analysis (Table 4) and the actions required to mitigate the possible failures that may occur during the SILS procedure.

**Figure 14:**
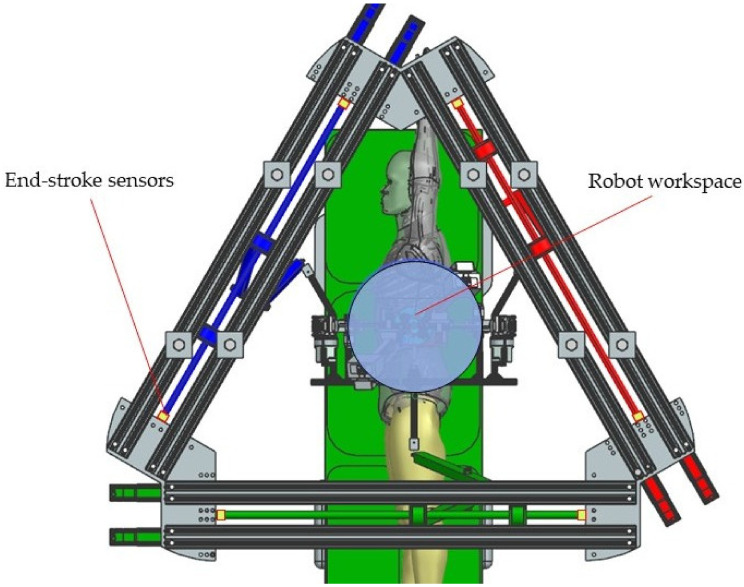
Robotic structure top of view with the workspace and the stroke sensors.

To prevent the F1 and F2 failure modes, the robot workspace has been simulated and studied, and the range of motion (length) of the linear screws is selected. A set of proximity sensors are placed on each end of the linear axes (Figure 14).

**Figure 15:**
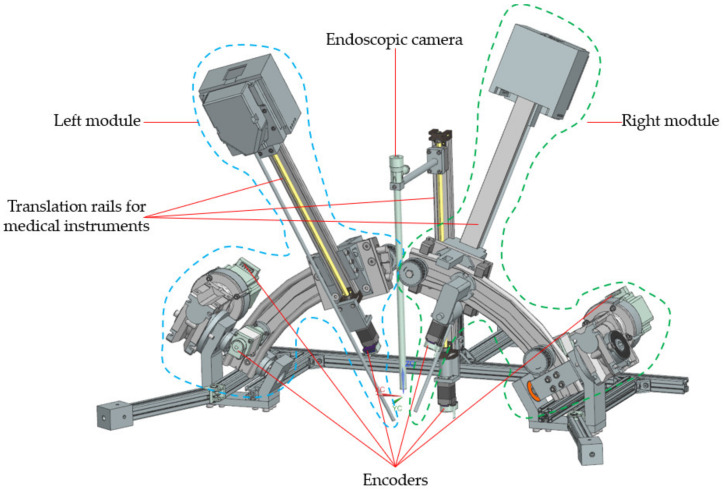
Mobile platform with all the medical instruments required for the surgery.

To prevent F1 and F2 in the case of the instruments guiding modules, a set of proximity sensors is installed at each end of the module tracks attached to the mobile platform (Figure 15).

**Figure 16:**
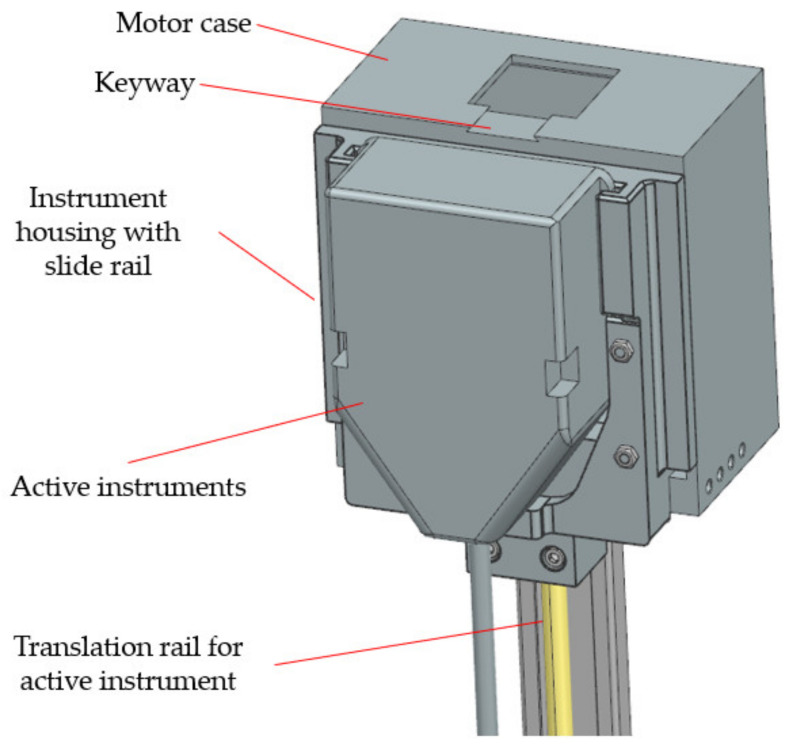
Medical instrument attachment to the guiding device.

Failure 3 is prevented during the design phase by using an adaptable casing that allows attaching a wide variety of instruments, which are locked using a keyway (Figure 16). This adaptable casing ensures that the instruments are securely attached and prevents any potential failure or detachment during the operation, thus enhancing the overall reliability and safety of the robotic system.

To prevent F4, a redundant sensor system is used, the homing position of the robotic structure is defined in the control unit, and the motors are equipped with encoders. The functionality of the robotic system is also checked in the pre-planning stage.

To prevent F5, a special subroutine is implemented in the control system that uses another communication protocol defined in the control architecture and connection to an external power supply is also used.

### 3.3. Results Regarding the Experimental Tests for the Instruments RCM Displacement Risk (MH3)

**Figure 17:**
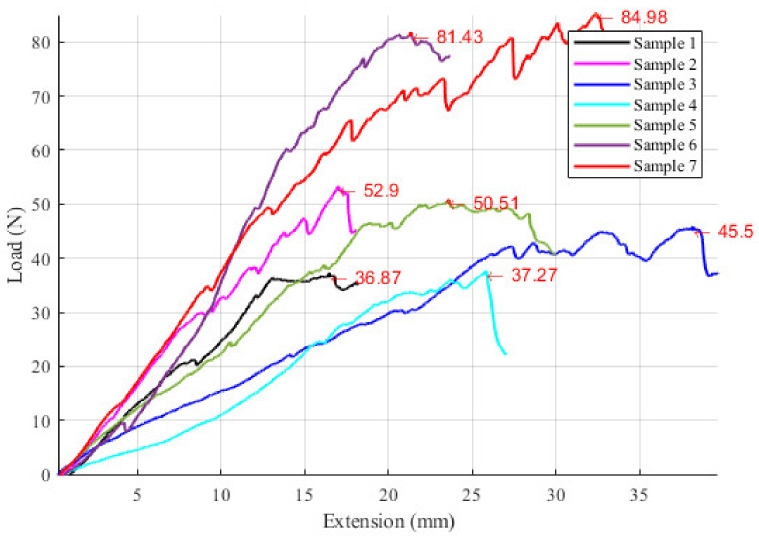
The force–extension graph of the studied pork tissue specimens.

The testing experiment results are presented in Figure 17, with the maximum registered force indicated in each sample testing. The plot shows the relation between the tissue elongation and the registered force, which can be further used to assess the influence of the two instruments’ RCM_S_ displacement on the patient’s tissue (what force is applied on the tissue according to the measured displacement). The registered maximum load is between 36.87 and 84.98 N (the moment when the tissue breaks off from the ribs), with a mean value of 55.64 N and a median of 50.51 N. The tissue extension recorded for the maxim corresponding forces applied to it ranges from 16.45 to 38.16 mm, with a mean value of 24.95 mm and a median of 23.57. The data present large variations, mainly due to the tissue thickness variation and, to a smaller extent, to tissue extension speed, which means that for safety reasons, the minimum values of tissue extension (and the corresponding maximum recorded force values) are further considered in this specific case.

**Figure 18:**
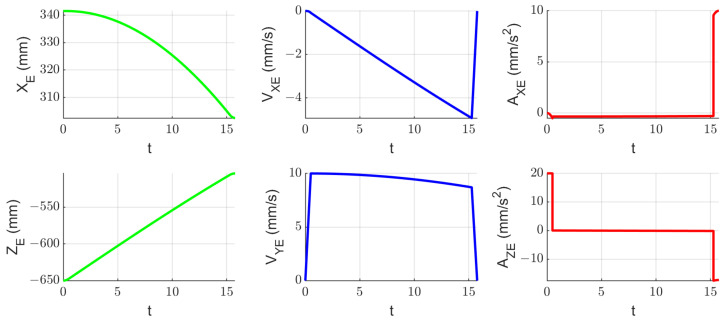
Time history diagram of the laparoscope tip (X and Z coordinates) during its orientation around the OY axis in terms of position (the green line), velocity (the blue line), and acceleration (the red line).

**Figure 19:**
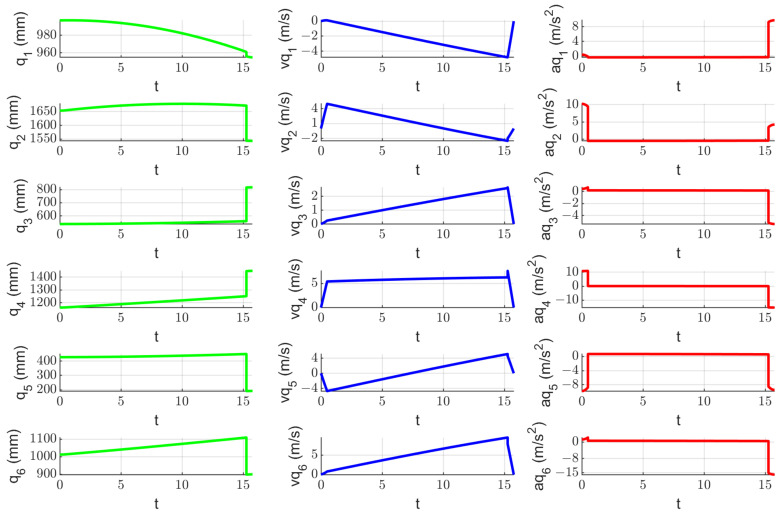
Time history diagram of the active joints of the 6—DOF parallel robot used to orient the laparoscope in terms of position (the green line), velocity (the blue line), and acceleration (the red line).

**Figure 20:**
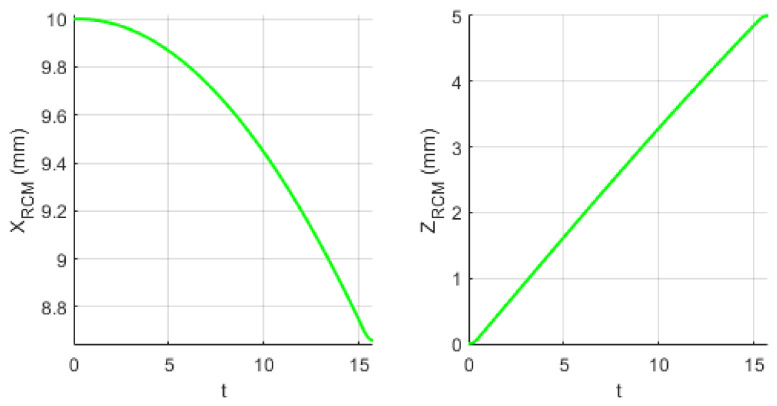
The SILS instruments RCM displacement during the laparoscope orientation on the OX and OZ axes.

A kinematic simulation of the robotic system to determine the tissue instruments’ RCM displacement during the laparoscope orientation has been performed. The orientation of the endoscopic camera from a vertical position (where the X and Y coordinates of the tip of the laparoscope and of the laparoscope RCM are identical—XB = 50 mm, YB = 300 mm) with a radius of R = 150 mm up to an angle of 30 degrees, which is the maximum estimated orientation angle according to [22,29], has been considered. The motion is performed in the OXZ plane, so the laparoscope rotates around the OY axis. The motion parameters at the tip of the instrument are: *v_max_* = 10 mm/s and *a_max_* = 20 mm/s2. These motion parameters lead to a value of ~20 mm/min tissue displacement at the level of the SILS instruments RCMs, considering d = 10 mm as the distance between the laparoscope RCM and the SILS instruments RCMs. Figure 18 presents the time history diagram of the laparoscope tip in terms of position, velocity, and acceleration, while Figure 19 presents the time history diagram of the 6-DOF parallel robot coordinates (q1–q6) during this simulation. Figure 20 shows the RCM displacement of one of the SILS instruments during the laparoscope orientation. The displacement recorded on the OZ axis is ~5 mm for one instrument, which means that the total tissue elongation is of ~10 mm, which is the maximum displacement considering that the laparoscope orientation range of 30 degrees exceeds the actual expected values for an SILS procedure.

### 3.4. Finite Element Analysis Results for the Main Component of the PARA-SILSROB System

A set of Finite Element Analyses (FEAs) were generated to validate the robot’s mechanical architecture. These analyses were carried out on the two main components of the robotic structure.

**Figure 21:**
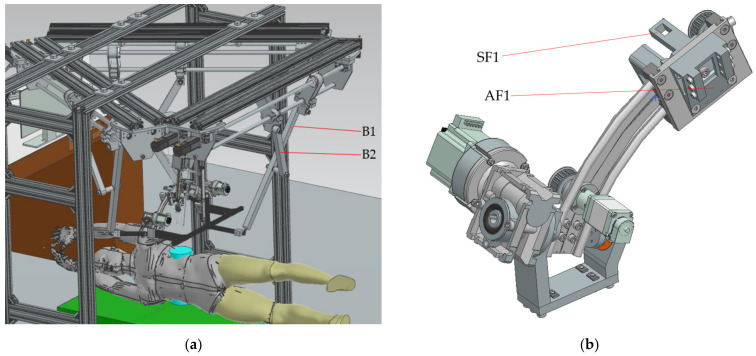
The main components of the robotic structure with the highest mechanical stresses (robotic structure (**a**) and module of the mobile platform (**b**)).

**Figure 22:**
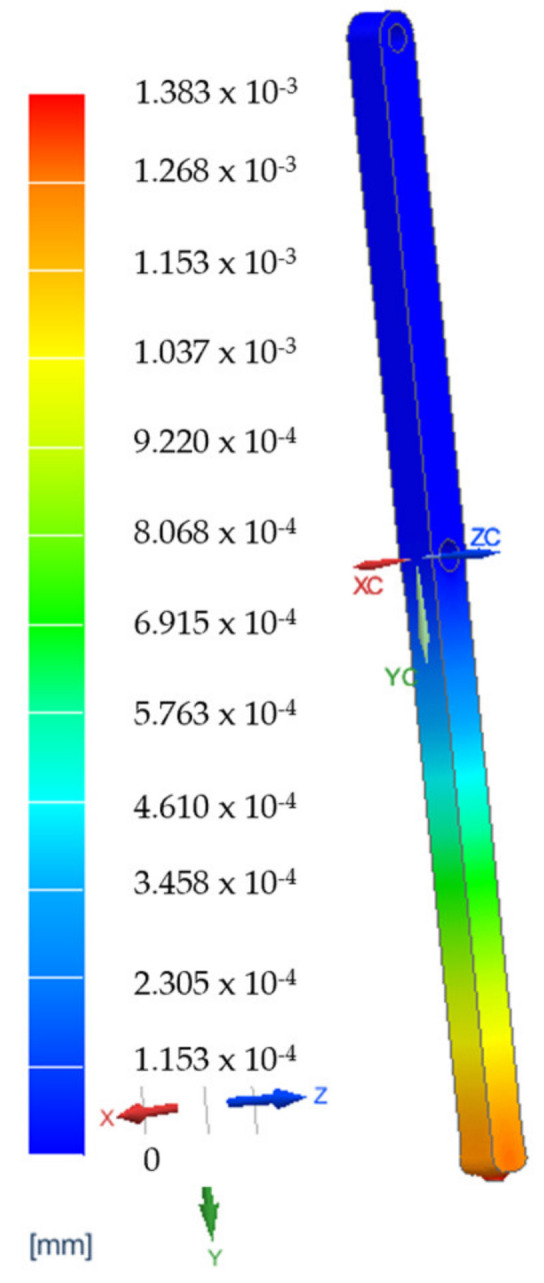
FEA results for element B1.

**Figure 23:**
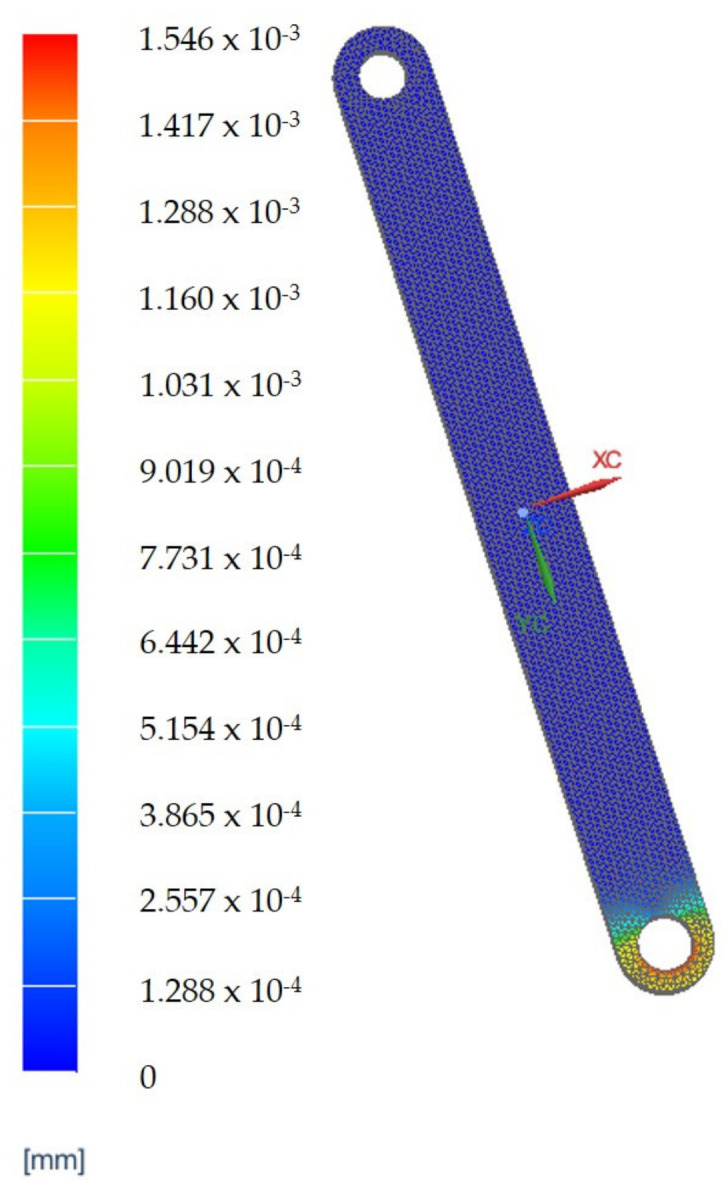
FEA results for element B2.

**Figure 24:**
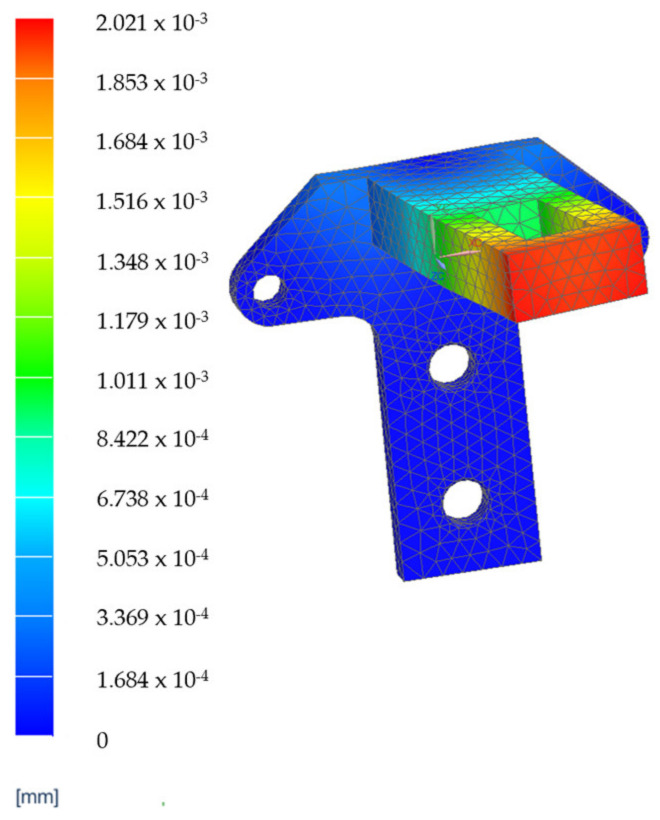
FEA results for element SF1.

**Figure 25:**
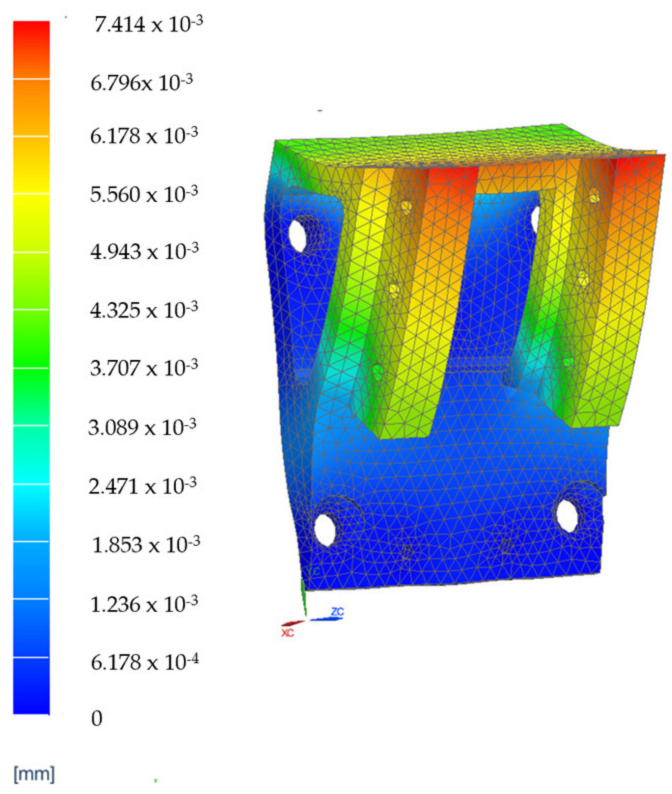
FEA results for element AF1.

The elements which are estimated to be the most mechanically stressed are the main arm, B1, the secondary arm, B2, of the robotic structure (Figure 21a) and steel flange, SF1, and the aluminum flange used for attaching the rail that performs the translation of the active instrument—AF1 (Figure 21b). The results of the FEA applied to these component elements are presented in Figure 22, Figure 23, Figure 24 and Figure 25.

The results of the finite element analysis are generated by applying static forces ranging from 300 (for the MP) to 500 Newtons (for the 6—DOF robot). The FEA shows negligible deformation values (B1—0.00138 [mm], B2—0.00154 [mm], SF1—0.00202 [mm], AF1—0.00741 [mm]), thus eliminating elasticities of the elements of the PARA-SILSROB robotic system, with direct influence upon the accuracy of the robot and thus the safety of the patient.

## 4. Discussion

This paper targets the design of a robotic system for SILS procedures that is able to successfully fulfill the medical procedure requirements in terms of safety and efficiency.

The technical specifications of a robotic system for SILS derived from the medical tasks requirements supplied by the medical expert’s team have been analyzed using the AHP method. Based on the medical task and the resulting technical characteristics, the concept of the robotic system has been designed. Thus, a 6-DOF parallel robot to guide a mobile platform hosting three modules has been proposed. One module has been designed o guide the laparoscope, the other two aiming to guide the active instruments. A parallel architecture, renowned for its stiffness, has been used to guide the MP, while the three modules provide a compact solution to fit the scarce space available within most operating rooms. The instruments guiding modules have an architecturally constrained RCM, which is of great importance regarding the accuracy of instrument positioning within the operating field. The kinematics of the mobile platform considers the RCM of the laparoscope as the mobile coordinate system, which can be further used within the compensation algorithm of the instruments’ RCM displacement during the laparoscope orientation.

The PARA-SILSROB design for safety is performed through a risk assessment based on ISO14971:2019 related to medical device regulations. A hazard identification and analysis based on a series of questionnaires completed by engineers and medical experts has been performed to determine the occurrence and severity. The outcomes have been considered in the design stage, aiming for their removal or mitigation.

The use of the FMEA method to identify possible failure modes within the functionality of the robotic system has pointed out several types of failures and their sources, which have led to various strategies proposed to avoid them. This has various benefits: it represents a cost-effective approach to prevent possible errors before the prototype development and prevents undesired and chaotic robot behavior due to various malfunctions.

Nevertheless, the proposed design has introduced a series of risks, one of them consisting in an overstrained tissue in the SILS port area when orienting the laparoscope. To evaluate this risk and estimate the possible negative effects, an experimental test using porcine tissue has been conducted. In the worst-case scenario (Sample 1), the maximum tissue displacement according to the simulation is 10 mm, corresponding to a load of approx. 25 N, according to the force–load graph presented in Figure 17. This value is less than 30% of the maximum tissue displacement at the rupture moment, which occurred for this sample at 36.87 N. The obtained results validate the proposed concept since no major negative side effects have been detected regarding the stress to which the patient’s tissue is subjected to during the laparoscope orientation within the range of ±30°.

The mechanical resilience of the robotic system components has been studied using Finite Element Analysis. The most stressed robotic system links and their behavior to specific loads, like the ones recorded during the SILS task performance, are presented. The results are positive and prove that the experimental model can be built based on the proposed design.

Based on the obtained results, the authors consider that the high stiffness coupled with versatility and large workspace qualify PARA-SILSROB as a good candidate for a successful robotic system for SILS. Compared to the commercially available robotic architectures, PARA-SILSROB can be easily used for various organ treatments (e.g., hepatocellular carcinoma, stomach cancer) using the SILS approach.

## 5. Conclusions

This paper focuses on the safety design of a new robotic system used in SILS. Starting from the medical protocol for SILS, the technical requirements are identified and prioritized, leading to the initial concept. Safety issues are identified using several tools like risk assessment and FMEA, and mitigation solutions are proposed. The obtained results and the actions taken to mitigate the risks were presented along with the results of the experimental tests, which combined with the finite element analyses of the main components of the robotic structure, validated the design of the robotic structure.

Future studies imply further development of the experimental model and laboratory tests using ex vivo porcine tissues.

## 6. Patents

Pisla, D., Birlescu I., Vaida C., Tucan P., Gherman B., Plitea N., Family of modular parallel robots with active translational joints for single-incision laparoscopic surgery, OSIM A00733/03.12.2021, 3 December 2021 [40].

## Figures and Tables

**Table 1:** The technical characteristics of the robotic structure for SILS.

Codification	Technical Characteristics
TC1	Safety. The robotic-assisted SILS procedure needs to be performed in completely safe conditions for the patient.
TC2	Versatility. Although the SILS procedure is usually limited to the umbilicus area, it should be compliant with other approaches as well. In this paper, the robotic proposed concept targets kidney treatment using the retroperitoneal approach.
TC3	Emergency compliance. The system and its components (instruments, modules) must be quickly removable from the operation site in case of emergency.
TC4	Stiffness. Since stiffness is closely related to the positioning accuracy of surgical instruments, a stiff architecture and mechanical structure is desirable.
TC5	Fast initialization procedure. A setup time of 25–35 min is specified in [28,31,32]. Placing the SILS instruments into a commercially available SILS port designed for manual intervention is not always an easy task, which is why the system should be fitted with additional devices to speed up the setup time.
TC6	Sterilization. The instruments need to be easily sterilizable, while the non-sterile equipment should be isolated from the operating field.
TC7	Collisions avoidance. The robotic systems modules should not collide during the intervention. Instruments collision might be inevitable, but the surgeon should be notified regarding this event.
TC8	Fit into the operating room. The robotic system should fit into the existing operating rooms, considering the other medical equipment required in a SILS procedure.
TC9	Tremor management. The tremor recorded at the tip of the SILS instruments has various sources. One of them is the natural vibration of the robot during the SILS instrument positioning. This aspect should be controlled well during the design phase.
TC10	Reachability. The tip of the medical instruments should be able to reach the lesion area with accuracy. This further means that the insertion length of the instruments should not negatively affect the positioning accuracy and the motion resolution [22,28].

**Table 2:** Severity score recorded for the PARA-SILSROB robotic system.

Hazard	E1	E2	E3	E4	E5	E6	E7	E8	E9	E10	Mean Value
MH1	98	100	98	100	99	100	90	96	98	100	97.9
MH2	93	98	95	98	96	90	98	99	100	90	95.7
MH3	97	100	100	90	98	95	100	98	95	98	97.1
EH1	95	90	95	95	90	95	90	90	98	94	93.2
EH2	99	99	96	98	95	96	98	100	98	95	97.4
EH3	55	70	55	65	40	45	55	35	60	55	53.5
EH4	80	90	80	75	85	70	75	60	70	70	75.5
EH5	95	95	97	94	98	95	95	98	95	97	95.9
TH1	96	90	95	97	95	85	98	95	90	95	93.6
TH2	90	95	80	88	89	95	85	90	87	95	89.4
VH1	99	95	100	97	95	90	95	99	95	90	95.5
NH1	15	55	25	35	20	25	35	25	40	20	29.5

**Table 3:** Probability score recorded for the PARA-SILSROB robotic system.

Hazard	E1	E2	E3	E4	E5	E6	E7	E8	E9	E10	Mean Value
MH1	60	60	70	70	60	55	60	55	65	60	61.5
MH2	70	65	60	55	70	60	60	55	70	50	61.5
MH3	60	65	55	60	65	75	65	65	60	60	63
EH1	45	65	50	45	35	45	55	45	60	55	50
EH2	25	10	35	35	30	35	20	20	35	45	29
EH3	15	20	10	20	15	20	10	15	10	15	15
EH4	15	30	15	25	15	25	25	35	20	25	23
EH5	45	30	35	25	20	35	40	30	25	20	30.5
TH1	60	50	55	60	55	60	65	55	50	70	58
TH2	25	20	15	30	35	20	20	25	30	25	24.5
VH1	25	20	30	35	45	35	45	20	35	30	32
NH1	20	10	20	25	10	10	10	20	25	30	18

**Table 4:** Failure Mode and Effects Analysis (FMEA) for PARA-SILSROB structure.

Function	Potential Failure Mode	Hazard Potential	Potential Cause of the Failure	Safety Action	Codification
Positioning	Wrong manipulation Reach the maximum range of motion	Possible patient injury Surgery failing System damage	Lack of end-stroke limiters Flawed system manipulation Wrong RCM definition	Mount end-stroke limiters Check the system functionality before performing the surgery (pre-planning)	F1
Orientation	Wrong manipulation Reach the maximum range of motion	Possible patient injury Surgery failing System damage	Lack of end-stroke limiters Flawed system manipulation	Mount end-stroke limiters Check the system functionality before performing the surgery (pre-planning)	F2
Instruments attach/detach	Attach/detach mechanism does not work properly	Possible patient injury	Failure of the mechanical interface	Design of a reconfigurable case equipped with a locking system for the instruments	F3
Sensor system	Incorrect feedback	Possible patient injury Incorrect robot motion	Sensor’s malfunction Encoder failure Power outage	Redundant sensor system Check the system functionality in the pre-planning stage Implement the homing position in the control unit	F4
Control system	Delays between the master console and the slave system Communication errors based on protocol communication failing	Possible patient injury Incorrect robot motion Surgery failing	Power supply failure Processor unit failure Wrong programming or failure communication system	Safety loop control Error tracking using an AI agent	F5

**Table 5:** Risk evaluation of identified hazards for the PARA-SILSROB robotic system.

Hazard	Score	Evaluation Score
MH1	159.4	High
MH2	157.2	High
MH3	160.1	High
EH1	143.2	Medium
EH2	126.4	Medium
EH3	68.5	Low
EH4	98.5	Low
EH5	126.4	Low
TH1	151.6	Medium
TH2	113.9	Medium
VH1	127.5	Medium
NH1	47.5	Negligible

**Table 6:** Risk evaluation of identified hazards for the PARA-SILSROB system.

Hazard	Risk Reduction Method
MH1	Design the laparoscope and instrument’s guiding modules to reduce the collision probability. Mounting the proximity sensors on the robot structure in areas with higher collision probability. Implementation of software limitation for the range of motion from the control unit. Monitoring in real time the position of each arm using the data from the motor encoders.
MH2	Definition of motion limits for the orientation of the laparoscope. Monitor the orientation angles of the platform and define alert messages for the surgeon.
MH3	Experimental tests have shown that the tissue damage due to the instrument RCM displacement is very low.
EH1	Use a generator or a UPS power supply.
EH2	Use low-voltage components. Reduce the direct contact between the patient and components under voltage.
EH3	Use of circuits with short circuit protection. Use of electric fuses.
EH5	Use secondary sensor system. Test the sensor system functionality before the medical procedure.
EH6	Define two different communication protocol.
TH1	Define a special motion when the hook electrode is used. Implement motion scaling in the control unit.
TH2	Actuating the instruments using cables. Limit contact of heat-generating parts with the patient body.
VH1	The reduction in vibrations in the robotic structure during the design phase using Finite Element Analysis (FEA) to determine the deformation of the main components.
NH1	Cover the robotic structure with noise-reducing material.

## Data Availability

Data sharing is not applicable to this article.

## Appendix A. Questionnaire

**Table A1:** How would you rate the following hazards in terms of severity? (The evaluation will be done by assigning scores to numbers between 0–100 for each hazard.).

		Catastrophic [100]	Serious [90–99]	Moderate [30–89]	Minor [0–29]
MH1	Robotic arms crushing due to wrong motion of the linear actuating joints (this hazard can be created if the robot is wrongly programmed)				
MH2	Cuts/scratches caused by wrong insertion of the instruments and the endoscopic camera in the patient body (this hazard can be created through incorrect definition of the insertion point in the patient’s body or incorrect assessment of the patient’s anthropomorphic characteristics)				
MH3	Impact between the mobile platform and the patient body (this hazard can be created if the surgeon wrongly operates the master console)				
MH4	The mechanical locking of modules used for handling active instruments or the mobile platform when the endoscopic camera is reoriented (this hazard can be created by incorrect control of the master console or improper programming of the robotic system)				
MH5	Impact between the active instruments and the endoscopic camera (this hazard can be created due to a malfunction of orientation modules attached to the mobile platform or improper handling of active instruments by the surgeon using the master console)				
MH6	Locking of the active translational joints (this hazard can be created by overloading of the robot structure or master console malfunction)				
MH7	Over the limit movements of the robot arms and the mobile platform (this hazard can be created if the robotic system is wrongly teleoperated)				
MH8	Cuts and scratches caused by edges of the metal parts (this hazard can be created by faulty design and manufacturing of the robotic structure and may occur when the robot is sterilized)				
EH1	Risk of power outage.				
EH2	Risk of electrocution of the patient.				
EH3	Motors in overload.				
EH4	Risk of short circuit.				
EH5	Risk of sensors system malfunction.				
EH6	Risk of communication protocol malfunction.				
TH1	Tissue burns caused by wrong manipulation of active instruments (Permanent Cautery Hook).				
TH2	Burns caused by overheating of the active instruments (Permanent Cautery Hook).				
VH1	Patient harm by robot structure entry in singularity positions.				
VH2	Patient harm caused by uncontrolled vibrations of active instruments and the endoscopic camera generated by malfunctions of the modules attached to the mobile platform or overloading of the active instruments.				
NH1	Acoustic discomfort caused by driving the motion of the mobile components when the robotic system is operated.				
ERH1	Risk to the medical act failing (this hazard can be created when the surgeon is improperly positioned at the master console)				

**Table A2:** How would you evaluate the probability of the hazard occurring in the case of the PARA-SILSROB robot structure? (The evaluation will be done by assigning scores to numbers between 0–100 for each hazard.).

		Very Likely [100]	Likely [90–99]	Unlikely [30–89]	Remote [0–29]
MH1	Robotic arms crushing due to wrong motion of the linear actuating joints (this hazard can be created if the robot is wrongly programmed)				
MH2	Cuts/scratches caused by wrong insertion of the instruments and the endoscopic camera in the patient body (this hazard can be created through incorrect definition of the insertion point in the patient’s body or incorrect assessment of the patient’s anthropomorphic characteristics)				
MH3	Impact between the mobile platform and the patient body (this hazard can be created if the surgeon wrongly operates the master console)				
MH4	The mechanical locking of modules used for handling active instruments or the mobile platform when the endoscopic camera is reoriented (this hazard can be created by incorrect control of the master console or improper programming of the robotic system)				
MH5	Impact between the active instruments and the endoscopic camera (this hazard can be created due to a malfunction of orientation modules attached to the mobile platform or improper handling of active instruments by the surgeon using the master console)				
MH6	Locking of the active translational joints (this hazard can be created by overloading of the robot structure or master console malfunction)				
MH7	Over the limit movements of the robot arms and the mobile platform (this hazard can be created if the robotic system is wrongly teleoperated)				
MH8	Cuts and scratches caused by edges of the metal parts (this hazard can be created by faulty design and manufacturing of the robotic structure and may occur when the robot is sterilized)				
EH1	Risk of power outage.				
EH2	Risk of electrocution of the patient.				
EH3	Motors in overload.				
EH4	Risk of short circuit.				
EH5	Risk of sensors system malfunction.				
EH6	Risk of communication protocol malfunction.				
TH1	Tissue burns caused by wrong manipulation of active instruments (Permanent Cautery Hook).				
TH2	Burns caused by overheating of the active instruments (Permanent Cautery Hook).				
VH1	Patient harm by robot structure entry in singularity positions.				
VH2	Patient harm caused by uncontrolled vibrations of active instruments and the endoscopic camera generated by malfunctions of the modules attached to the mobile platform or overloading of the active instruments.				
NH1	Acoustic discomfort caused by driving the motion of the mobile components when the robotic system is operated.				
ERH1	Risk to the medical act failing (this hazard can be created when the surgeon is improperly positioned at the master console)				
